# Translating comparative effectiveness of depression medications into practice by comparing the depression medication choice decision aid to usual care: study protocol for a randomized controlled trial

**DOI:** 10.1186/1745-6215-14-127

**Published:** 2013-05-07

**Authors:** Annie LeBlanc, Amy E Bodde, Megan E Branda, Kathleen J Yost, Jeph Herrin, Mark D Williams, Nilay D Shah, Holly Van Houten, Kari L Ruud, Laurie J Pencille, Victor M Montori

**Affiliations:** 1 Department of Health Sciences Research, Division of Health Care Policy and Research, Mayo Clinic, Rochester, MN, USA; 2 Knowledge and Evaluation Research Unit, Mayo Clinic, Rochester, MN, USA; 3 Center for the Science of Healthcare Delivery, Mayo Clinic, Rochester, MN, USA; 4 Department of Health Sciences Research, Division of Biomedical Statistics and Informatics, Mayo Clinic, Rochester, MN, USA; 5 Yale University School of Medicine, New Haven, CT, USA; 6 Department of Psychiatry and Psychology, Mayo Clinic, Rochester, MN, USA; 7 Department of Medicine, Division of Endocrinology, Mayo Clinic, Rochester, MN, USA; 8 Health Research & Educational Trust, Chicago, IL, USA

**Keywords:** Depression, Comparative effectiveness research, Shared decision making, Decision aid, Implementation, Randomized controlled trial

## Abstract

**Background:**

Comparative effectiveness research (CER) documents important differences in antidepressants in terms of efficacy, safety, cost, and burden to the patient. Decision aids can adapt this evidence to help patients participate in making informed choices. In turn, antidepressant therapy will more likely reflect patients’ values and context, leading to improved adherence and mood outcomes.

**Methods/Design:**

The objective of this study is to develop the Depression Medication Choice decision aid for use during primary care encounters, and to test its efficacy by conducting a clustered practical randomized trial comparing the decision aid to usual depression care in primary care practices.

We will use a novel practice-based, patient-centered approach based on participatory action research that involves a multidisciplinary team of designers, investigators, clinicians, patient representatives, and other stakeholders for the development of the decision aid. We will then conduct a clustered practical randomized trial enrolling clinicians and their patients (n = 300) with moderate to severe depression from rural, suburban and inner city primary care practices (n = 10). The intervention will consist of the use of the depression medication choice decision aid during the clinical encounter. This trial will generate preliminary evidence of the relative impact of the decision aid on patient involvement in decision making, decision making quality, patient knowledge, and 6-month measures of medication adherence and mental health compared to usual depression care.

**Discussion:**

Upon completion of the proposed research, we will have developed and evaluated the efficacy of the decision aid depression medication choice as a novel translational tool for CER in depression treatment, engaged patients with depression in their care, and refined the process by which we conduct practice-based trials with limited research footprint.

**Trial registration:**

Clinical Trials.gov: NCT01502891

## Background

Depression is one of the most common chronic illnesses in the USA, and decreases the quality and duration of life of over 16 million Americans. Impacting not only individuals, depression causes suffering to family and friends, losses in productivity, and other costs to society [1]. Despite a lifetime prevalence of 17%, depression is often undetected and suboptimally managed, particularly in primary care where 80% of patients with depression receive care and 75% of antidepressant prescriptions are provided [2,3]. Unfortunately, adherence to antidepressants is low, with contemporary cohorts citing 6-month rates in the range of 13% to 60% [4,5], and many patients who are initially adherent discontinue prematurely [6]. Primary nonadherence and premature discontinuation are associated with 77% increased risk of relapse or recurrence, and adverse effects on disability and healthcare utilization [7-9]. Thus, even when patients with depression are detected and diagnosed properly, over 60% will have adequate fidelity to treatment and many will suffer protracted clinical course with great cost implications in terms of dollars and human suffering.

Lifestyle changes, self-care practices, evidence-based psychotherapy and pharmacotherapy can improve depression outcomes, lower healthcare costs and improve the quality of life. When it comes to pharmacotherapy, depression care often demands primary care clinicians’ and patients’ use of therapy and medication regimens of different efficacy, safety, cost, and burden to the patient; findings vary according to depression severity and recurrence, underscored by the emerging literature on their comparative effectiveness [10,11]. Moreover, the translation of the evidence from comparative effectiveness research (CER) remains limited [12].

Patient decision aids can facilitate the translation of CER into practice. When systematically summarized, CER can inform the content of decision aids, including the range and likelihood of desirable and undesirable outcomes across the different options. These tools can be designed to facilitate patient involvement in making decisions even among patients and clinicians with limited experience in shared decision making [13-15]. We have previously shown that decision aids that provide easily accessible information about treatment options and their outcomes for other chronic conditions facilitated knowledge transfer and greater patient involvement in usual primary care settings, and showed promise in improving adherence to therapy [14,16,17]. To our knowledge, there is no research evaluating the use of patient decision aids within the clinical encounter, to facilitate the participation of patients with moderate to severe depression in choosing antidepressants in practice.

Consequently, to determine the ability of a decision aid to effectively translate CER about antidepressants into practice, we will first develop a decision aid, the Depression Medication Choice, for use during primary care encounters that would meet the needs of key stakeholders (patients and clinicians, health plans, and payers), while enabling shared decision making as a way of translating antidepressant CER into practice. We will then enroll primary care practices and randomly assign these to either usual depression care or to care involving use of the depression medication choice decision aid*.* This trial, still underway, will generate preliminary evidence of the relative impact of the decision aid on patient involvement in decision making, decision making quality, patient knowledge, and 6-month measures of medication adherence and mental health compared to usual depression care.

## Methods/Design

### Development of the Depression Medication Choice decision aid

**Figure 1:**
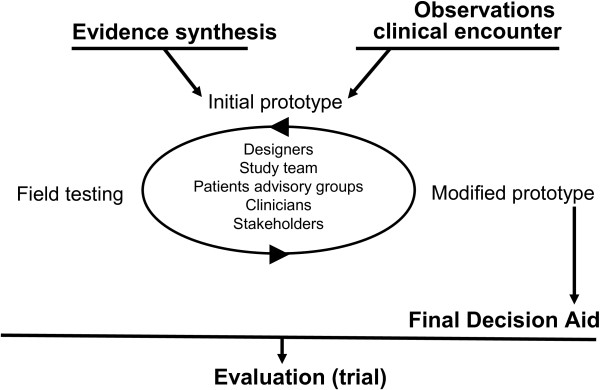
Development of a decision aid.

We will use a novel practice-based, patient-centered approach for decision aid development based on design/participatory action research, developed and validated by the Knowledge and Evaluation Research Unit at Mayo Clinic (Rochester, MN, USA), and executed by a multidisciplinary team comprised of designers, patients, primary care and psychiatry clinicians, and decision making and health literacy scientists (Figure 1) [13,14,17].

This approach includes three main steps: 1. synthesis of clinical evidence regarding the risks and benefits of each option, and its approval by stakeholders; 2. observations of real-time clinical encounters involving clinicians and their patients with depression facing the decision to use antidepressants; and 3. prototyping of the decision aid through a series of iterations and field testing in the same context as the initial observations.

#### Synthesis of evidence

For the purpose of the development of the depression medication choice decision aid, we will synthesize the evidence regarding the risks and benefits of antidepressants based on the Agency for Healthcare Research and Quality (AHRQ) effective healthcare systematic review report on the comparative effectiveness of second generation antidepressants (07-EHC-007) [18]. We will then convene psychiatrists, primary care clinicians, patients and health plan representatives to meetings in which they will review the summary, and agree on its results and associated credibility and applicability.

#### Observation of encounters

Members of the research team led by a designer will observe approximately 10 to 15 usual clinical encounters, typically 3 to 5 without aids, and 3 to 5 with each iteration of the decision aid prototype. Using a predefined observation grid we developed and used in previous studies, we will look for patterns of patient-clinician conversations regarding antidepressant medications, as well as nonverbal behaviors and attitudes.

#### Prototyping and field testing

The construction of the Depression Medication Choice will be based on the summary of evidence and clinical observations, guided by the International Patient Decision Aid Standards (IPDAS) [19], which provides recommendations and standards for the development of decision aids. The initial prototype of the decision aid will result from discussions with members of the study team, clinicians, and patients from our patient advisory groups. They will be asked to comment on the content, format and ease of use of the prototype, on how the prototype aid captures current knowledge and addresses their concerns about antidepressants, and on to what extent the prototype helps bring to light patient context, values and preferences for decision making. Once developed, we will pilot this initial prototype in 3 to 5 clinical encounters, looking for patterns of the conversations, and documenting the issues, problems and challenges. When feasible, we will conduct a brief structured interview with the patient and the clinician after the encounter. The study team will then evaluate the quality of the conversations and the ability of the prototype to facilitate the decision making process. This process will be repeated with subsequent prototypes until the research team and stakeholders reach a consensus that the prototype is successful in involving patients in decision making (in our prior work it has taken up to seven iterations). This process should result in a discrete and highly intuitive decision aid, requiring minimal training to use, and designed to meet the needs of patients and their primary care clinicians working in fast-paced care settings.

### Evaluation of the impact of the Depression Medication Choice decision aid

#### Study design and setting

We will conduct a clustered, practice-based, randomized trial of the Depression Medication Choice decision aid in ten rural, suburban and inner city primary care practices in the Midwest USA. The Mayo Clinic Institutional Review Board (IRB) has approved the study procedures described herein. We also obtained ethical approval from Hennepin County Medical Center Human Subjects Research Committee for the three participating practices not covered under the Mayo Clinic IRB. The trial design and protocol are registered in ClinicalTrials.gov (http://NCT01502891).

#### Study participants

Primary care practices were invited to participate in the study based on key criteria for a successful collaboration identified from past similar trials: sufficient number of clinicians interested in participating; an easily identifiable clinical champion; a large enough patient population meeting our inclusion criteria; and an on-site, or willingness to have an on-site, study coordinator. Practices that had concurrent research studies involving antidepressants were automatically excluded from participating in the study.

Primary care clinicians (physicians, nurse practitioners, physician assistants) from participating practices will be eligible for the study if they provide care to patients suffering from depression. Eligible clinicians will be invited to participate in the study through an initial meeting held by the investigators at the practice or individually by the on-site study coordinator.

Adult patients will be eligible for the study if they have a diagnosis of depression as judged by the clinician, a score on the Patient Health Questionnaire (PHQ-9) ≥10 [20], receive their depression care in the primary care setting, do not have bipolar disorder (as judged by the clinician), and have no major barrier (language, sensorial, cognitive) to providing written informed consent.

Eligible patients will be identified in advance from the appointment schedules of clinicians. All eligible patients who present to the practice for a depression visit during the study will be invited by an on-site study coordinator to participate in the study.

#### Allocation procedures

We will randomly allocate practices to the intervention or usual care arm. We will pair practices by size (the number of clinicians seeing adult patients with depression) and by whether they have implemented the DIAMOND (Depression Improvement Across Minnesota, Offering a New Direction) program, a practice redesign initiative to improve depression care through the use of care coordinators [21]. Within each pair, we will randomize practices to either: 1. the use of Depression Medication Choice within the clinical encounter; or 2. usual care. A study statistician will perform the randomization centrally after the practices have been enrolled and ensure concealment of allocation.

Practices, clinicians and investigators will not be masked to allocation. However, patients will remain unaware of the study’s main hypotheses. To prevent loss to follow-up and support the intention-to-treat principle, we will centrally follow patients, and ensure that patient surveys and pharmacy follow-ups are complete.

#### Intervention

The intervention will consist of the use of the Depression Medication Choice decision aid by patients and their primary care clinician during the clinical encounter. Training of clinicians will be provided prior to enrollment of their patients and will include a demonstration showing how to use the decision aid with patients. The focal points of the demonstration will be: 1. the decision aid serves as a guide for conversation rather than a script; 2. clinicians have flexibility in the manner in which they use the decision aid, including how and when they use it during the visit; and 3. clinicians may elect not to use the tool with certain enrolled patients, per their own judgment. Brief video clips and a storyboard to demonstrate the basic use of the decision aid will be available for clinicians to review at their convenience. A study team member will remain available to undertake one-to-one demonstrations after the initial group demonstration, as needed.

#### Usual care

For patients in the usual care arm, clinicians will manage the discussion about medication regimen as usual, without using the decision aid.

#### Data collection and analysis

We will collect patients’ data through: 1. self-reported questionnaires administered before and after the clinical encounter with their clinician, and at 3 and 6 months post-encounter; and pharmacy data 2. information about depression-related care included in medical records. Participating clinicians will be given brief questionnaires to complete once before and then immediately following each clinical encounter with a participating patient. In addition, we will videotape each clinical encounter where consent to do so has been provided by both the patient and clinician. Patient, clinician and practice socio-demographic information will be collected through questionnaires, and administrative or patient medical records.

#### Outcomes measures

The RE-AIM framework, developed to address how an intervention is implemented in a real-world setting, will guide this portion of the study and includes the following dimensions: Reach (how broadly is this intervention used within the practices), Effectiveness (what is the impact of the intervention on outcomes), Adoption (can this be adopted by new groups with ease and minimal modifications), Implementation (what are the special issues and barriers in implementation), and Maintenance (can the intervention be maintained and will the impact continue) [22,23].

To assess the Reach of the decision aids, we will use a tracking log to record patients who are enrolled as well as those who declined the invitation to participate, and their respective characteristics. This will allow us to measure participation and representativeness.

To assess the Effectiveness of the decision aids, we will measure patient decisional conflict as the primary outcome, and decision making quality and processes (that is knowledge, satisfaction, preference in decision making style, patient involvement in decision making), depression outcomes, and medication adherence as secondary outcomes in the following ways:

1 Decisional conflict*.* Patients will complete the decisional conflict scale (DCS) immediately after the clinical encounter, the most commonly used outcome measure in decision aid trials [24,25]. Psychometric properties include good internal consistency, test-retest reliability, and effect sizes for responsiveness to change ranging from 0.4 to 1.2. Clinicians will also complete a similar scale immediately after the clinical encounter [26].

2 Knowledge*.* Patients will complete a questionnaire addressing general and specific knowledge about depression care contained in the decision aids. This questionnaire will be developed according to prior recommendations and will be completed after the encounter, and at 3 and 6 months post-encounter.

3 Satisfaction*.* Patients and clinicians will be asked questions regarding the acceptability and satisfaction regarding the way information was shared during the encounter.

4 Preference. Patients will identify their preference in decision making style prior to and after the encounter [27]. Clinicians will also complete the same questionnaire at enrollment in the study and following each encounter with a participating patient.

5 Patient involvement in decision making*.* We will use the Observing PatienT Involvement in shared decisiON making (OPTION) scale to assess the extent to which clinicians seek to engage patients in decision making [28]. Our group has extended the use of this third-observer tool using video recordings of the encounters, with excellent inter-rater reliability (intraclass correlation coefficient >0.7) [14,16,17,29].

6 Depression outcome*.* Patients will complete the nine items of the PHQ-9 [20], as a measure of the severity of depression symptoms, before their encounter, and at 3 and 6 months post-encounter.

7 Medication adherence*.* Patients’ measure of adherence and persistence to their depression medication will be derived from patient self-report and pharmacy records. We have been able to obtain complete pharmacy records for all of our participants in previous trials [14,16,17]. When mentioned the Adoption of the decision aids, we will estimate the proportion of practices or clinicians who adopt the intervention. Using clinician surveys and medical record review, we will compare the adoption rates across the intervention practices. We will select a random sample of participating patients’ medical records from each site to determine the extent to which the use of the decision aid is discernible in the records, and the extent of use and success when mentioned.

To assess the Implementation of the decision aid, we will seek to determine the extent to which the intervention (that is, use of the depression medication choice decision aid) is implemented as intended. Using the video recordings of the clinical encounters, we will assess the fidelity with which the decision aids are delivered and used as intended during these clinical encounters. The fidelity checklist is specific to the decision aid and setting, and will be developed based on previous checklists for other decision aids.

We will not be able to assess the Maintenance of the use of decision aid in practices as this requires a time period that is outside of the scope of the funding of the current study.

#### Socio-demographic and other patient characteristics

At entry in the study, patient characteristics collected will include age, gender, marital status, education, income, subjective health literacy [30,31] and subjective numeracy [32]. We will collect the following clinician characteristics: gender, birth year, position, practice specialty and number of years in practice. Practice-level characteristics will include distribution of patient ethnicity, race, payment method, population of community, location of clinic (urban, suburban, rural), distribution of full-time equivalent across staff (clinicians, nursing staff, educators), average number of visits per week and average length of appointments.

#### Sample size

The Statin Choice randomized trial evaluated the decision quality by comparing the decision aid to usual care, and reported a 9.8 point difference in decision quality with the standard deviation (SD) of 16.9 and 14.1 for the usual care and decision aid groups, respectively [16]. Thus, to determine the statistical power of the current trial, we made the following assumptions: 1. variances will be as reported in the statin choice study; 2. the trial should detect a difference of 9.8 points or greater in decisional conflict between two groups at a significance level of 0.05, with a two-sided t-test; 3. a modest correlation of outcomes within practices represented by an intracluster correlation coefficient (ICC) (between cluster variance/total variance) of 0.05; and 4. a 10% attrition rate. We estimated the power using a standard calculation for a t-test, adjusted for clustering by a variance inflation factor (VIF) (1 + (n - 1) x ICC), where n is the number of patients per practice [33]. Under these assumptions, we estimated that we will have 90% power to detect at least a 9.8 difference in the DCS if we are able to recruit 30 patients per clinic for a total recruitment target of 300 patients. Assuming a similar ICC and attrition rate for other outcomes, this sample size will have 99% power to detect 1 SD difference in any continuous measure (for example approximately a 2-point difference in a 10-question knowledge scale), and 80% power to detect a 30% difference in 6-month adherence rates assuming a control adherence rate of 50%. Actual power is likely to be greater because we will adjust for baseline values and characteristics, and because of our conservative assumptions.

### Analysis

Rather than assume that patient outcomes are independent of the clinician and practice, we will use techniques appropriate for clustered observations [33,34]. For unadjusted comparisons, we will use cluster-adjusted t-tests and chi-square tests, and for all adjusted analyses we will use hierarchical generalized linear models (HGLMs) [35],]. These statistical methods address the ‘unit of analysis’ issue through terms for each level of grouping or clustering, and have a number of features important to this study. Specifically, these models account appropriately for clustering of patients within clinicians and practices, and allow us to deal with repeated observations of patients. Three types of outcomes will be compared between randomization groups, as classified by measurement period and unit of observation: 1. patient-level after the index visit; 2. patient-level after 6 months; and 3. clinician-level outcomes at end of the study period. All patient and clinician outcomes will be analyzed as randomized in adherence to the intention-to-treat principle. Patient characteristics will be compared between randomization groups, and all outcomes adjusted for any that are not balanced between groups.

We will measure the following outcomes at the patient-level after the index visit: 1. Decision to initiate antidepressants. This will allow us to estimate the differences in prescription of medication for the decision aid group compared to the control group. As a secondary analysis, we will conduct subgroup analyses by health literacy level to test for an interaction. We will use this test to determine whether the difference in uptake of depression medications in the decision aid and control arms differs for the low versus high health literacy groups; 2. Decisional conflict and decisional quality and processes. Through these analyses, we will evaluate the effect of decision aids on decisional quality, patient satisfaction with the encounter, and patient knowledge related to the decision.

We will evaluate the following outcomes at the patient-level using 3- and 6-month data: 1. Antidepressant medication persistence and adherence based on decision at the index visit. Medication persistence will be measured as continuation of depression medications at 6 months for patients who leave the index visit with prescriptions for antidepressants; this includes their first medication and changes in the prescriptions that occur in the 6 months following the index visit. Medication adherence will be measured using fill dates from the pharmacies, measured using the percentage of days covered approach; 2. Depression outcomes. Depression treatment outcomes will be measured at baseline and at 3 and 6 months using the PHQ-9 [20]. This is the measure used by practice-based interventions in primary care, such as the DIAMOND initiative, there are practice pathways to collect this information, its score is linked to clinical action and it is familiar to primary care clinicians [36].

Finally, we will evaluate the clinician-level outcomes also using random effect models. However, for these analyses we will only cluster within clinic and account for the repeated measures for satisfaction and visit length, while controlling clinician and practice characteristics.

## Discussion

This study seeks to develop a decision aid for depression medication, to be used by clinicians and patients in the clinical encounter, and to evaluate its impact in a clustered randomized trial in diverse primary care practices. Our use of this tool is consistent with a strong theoretical and empirical basis supporting the use of decision aids, our preliminary work supporting the feasibility and efficacy of decision aids in other treatment decisions with patients with chronic conditions, and the evolving international, national and local policy support for patient involvement in decision making [14,16,17,37].

In our view, this protocol offers several innovative and important aspects. We propose the development and use of a novel translational tool in depression care, the Depression Medication Choice decision aid, to help clinicians and patients engage in meaningful conversations regarding the use of antidepressants. Moreover, we will be using the design/participatory action research we have used to design user- and context-sensitive decision aids. Furthermore, the design of the study reflects years of lessons learned while conducting practice-based trials in primary care settings aimed at minimizing research footprint in practices (see list below of key elements we find contribute to the successful implementation of practice-based decision aid trials).

### Key elements of conducting successful practice-based trials of decision aids

• Recruiting practices, rather than clinicians alone, so that every member of the staff is engaged in the conduct of the trial.

• Ensuring that targeted practices have both a clinical champion and sufficient eligible patients to meet recruitment goals.

• Ensuring the recruitment of clinicians with sufficient eligible patients within each practice.

• Involving practices, clinicians, patients and staff in the development of the intervention (decision aid), so that it meets their needs.

• Engaging clinicians and patients as collaborators of the study, rather than as ‘subjects’ of the study.

• Favoring an opt-out approach while recruiting clinicians.

• Minimizing research footprint on the practice (using study coordinators to identify and recruit patients, operating mindful of the practice workflow).

• Minimizing burden of the trial procedures on patients and clinicians with brief and few surveys, making paper and electronic surveys available, and using already scheduled meetings by the practice to provide study updates.

Finally, this proposal will help to highlight ways by which we can better engage patients with depression in their care. The evidence on shared decision making among patients receiving mental healthcare is still emerging [38-40]. Clinicians’ concerns about the ability of patients with mental healthcare needs to participate in healthcare decisions persists, and may be compounded by prejudices and misunderstandings regarding the capacity of these patients [39]. However, patients with mental healthcare needs have demonstrated competence in healthcare decision making [41], and the Institute of Medicine gives a strong recommendation for using decision support with all patients with mental healthcare needs regardless of the condition’s severity [42]. Additionally, patients with depression may experience additional benefits from the shared decision making process as they become engaged in their care, and find that their preferences and opinions are valued [39,43,44].

Upon completion of the proposed research we will have developed and evaluated the efficacy of the decision aid Depression Medication Choice as a novel translational tool for CER in depression treatment, and refined the process by which we conduct practice-based trials with limited research footprint.

### Trial status

Active recruitment.

## Abbreviations

AHRQ: Agency for Healthcare Research and Quality; CER: comparative effectiveness research; DCS: decisional conflict scale; DIAMOND: Depression Improvement across Minnesota, Offering a New Direction; HGLM: hierarchical generalized linear model; ICC: intracluster correlation coefficient; IPDAS: International Patient Decision Aid Standards; IRB: Institutional Review Board; OPTION: observing patient involvement in shared decision making; PHQ-9: Patient Health Questionnaire; RE-AIM: Reach Effectiveness Adoption Implementation Maintenance; SD: standard deviation; VIF: variance inflation factor.

## Competing interests

The authors of this protocol disclose no financial conflict of interest pertinent to this study. The Knowledge and Evaluation Research (KER) Unit at Mayo Clinic houses the processes of design and evaluation of decision aids, decides on topics of investigation, pursues funding, designs and conducts evaluation trials, and reports their findings. Investigators at the KER Unit, including authors of this manuscript, do not receive funding from any for-profit pharmaceutical or device manufacturer, nor do they receive any royalties or other monetary benefits, directly or indirectly, from the use of the decision aids. The KER Unit makes effective decision aids available online free of charge at http://shareddecisions.mayoclinic.org.

## Authors’ contributions

AL participated to the design of the study and wrote the first draft of the manuscript. AEB contributed to the first draft of the manuscript and made critical revisions to the manuscript. MEB contributed to the design of the study and to the first draft of the manuscript, and made critical revisions to the manuscript. KJY, MDW, JH and NDS contributed to the design of the study and provided revisions of the manuscripts. VMM conceived and designed the study, applied for funding, and made critical revisions to the manuscript. LJP, KLR and HVH provided revisions of the manuscript. All authors approved the final version of this manuscript.
